# Comparison between PRP and PRFM on FTSG healing profile: Macroscopic, microscopic and ELISA evaluation

**DOI:** 10.1016/j.amsu.2021.102350

**Published:** 2021-04-28

**Authors:** Mirta Hediyati Reksodiputro, Alida Roswita Harahap, Nurjati Chairani Siregar, Safarina Golfiani Malik, Jenny Bashirudin, Muhammad Thaufiq Siddiq Boesoirie, Dini Widiarni Widodo, Sandi Iljanto, Dondin Sajuthi, Lugyanti Sukrisman, Mikhael Yosia

**Affiliations:** a Department of Otorhinolaryngology-Head and Neck Surgery, Faculty of Medicine, Universitas Indonesia, dr. Cipto Mangunkusumo Hospital, Jakarta, Indonesia; b Department of Clinical Pathology, Faculty of Medicine, Universitas Indonesia, dr. Cipto Mangunkusumo Hospital, Jakarta, Indonesia; c Department of Pathology Anatomy, Faculty of Medicine, Universitas Indonesia, dr. Cipto Mangunkusumo Hospital, Jakarta, Indonesia; d Eijkman Institute for Molecular Biology, Jakarta, Indonesia; e Department of Otorhinolaryngology-Head and Neck Surgery, Faculty of Medicine, University of Padjajaran, Hasan Sadikin Hospital, Bandung, Indonesia; f Faculty of Public Health, Centre for Health Administration, Management and Policy, Universitas Indonesia, Jakarta, Indonesia; g Department of Clinics, Reproduction, and Pathology, Faculty of Veterinary Medicine, Institute Pertanian Bogor, Bogor, Indonesia; h Department of Internal Medicine, Faculty of Medicine, Universitas Indonesia, dr. Cipto Mangunkusumo Hospital, Jakarta, Indonesia

**Keywords:** PRP, PRFM, FTSG, Wound healing

## Abstract

**Background:**

Studies had shown the benefit of PRFM and PRP in wound healing but their use in skin graft healing was rarely studied. This study aims to compare the use of PRP and PRFM in accelerating wound healing process of skin full-thickness skin graft (FTSG).

**Materials and methods:**

Five pigs were used to look at the wound healing effect of PRP and PRFM usage prior to FTSG implantation. Subsequent punch biopsies were then conducted on the 1st, 3rd, 7th, 14th, and 30th day to obtain samples for macroscopic (skin color), extracellular matrix (collagen), microscopic (PMN, macrophage, and fibroblast), and ELISA (TGFβ1 and PDGF) analysis to determine the level of wound healing activity. ImageJ software was used to photograph for macroscopic and extracellular matrix analysis.

**Results:**

Macroscopic, extracellular matrix, and ELISA evaluation show no significant difference in FTSG survival rates for all treatment groups. Microscopic examination showed an increase in PMN, macrophage, and fibroblast levels with PRFM application showing higher increases in all observed microscopic variables compared to PRP and control.

**Conclusion:**

This study observed that both PRFM and PRP as autologous platelet preparation accelerate wound healing in FTSG, with PRFM being superior due to the higher number of PMN, macrophage, and fibroblast.

## Highlights

• Platelet-rich plasma (PRP) is a platelet-derived product used in wound healing.
• Platelet-rich fibrin matrix (PRFM) is an enhanced version of PRP believed to have superior quality in helping wound healing.
• Using PRFM in skin graft is convenient as it can be made into a sheet forms.
• PRFM used in skin graft showed superior wound healing properties shown via higher number of PMN, macrophage, and fibroblast compared to PRP.

## 1 Background

Skin grafts have been used since the 19th century and have been widely used in the surgical area. In modern times skin graft is commonly used in many surgical specialties including otolaryngology head and neck plastic reconstructive surgery. The skin graft is used for covering skin defects, reducing wound contraction, and accelerating wound healing that cannot be primarily healed. There are two categories of skin graft; full-thickness skin graft (FTSG) which consists of all layers of dermis and epidermis and split-thickness skin graft (STSG) which only consists of the epidermis and part of the dermis. FTSG had been known to be the ideal skin graft for the facial area [1].

Wound healing is an intricate process consisting of four major steps, hemostasis, inflammation, proliferation, and maturation. Thrombocyte plays an important role in releasing growth factors such as TGFβ1 and PDGF which further activates other cells including polymorphonuclear neutrophils (PMN), macrophage, and fibroblast. A study by Lucarelly et al. had shown that TGFβ1 and PDGF are essential in the initial phase of wound healing because of their role in recruiting PMN and macrophages [2]. This process naturally happens in the inflammation and proliferation stage of wound healing (which occurs within the first weeks), but the wound healing process will continue, with varying lengths based on the wound type. Delayed wound healing causes a significant clinical and economic burden by causing poor quality of life, prolonged hospital stays, and increasing need for post-discharge long term care [[2], [3], [4]].

Tissue engineering is a relatively new concept in which a combination of cell, scaffold, and growth factors was used to promote tissue regeneration. Tissue engineering development resulted in the discovery of biomaterial to accelerate wound healing process. One of the products is platelet-rich fibrin matrix (PRFM), which is a platelet-derived product. Before PRFM, platelet-rich plasma (PRP) was more commonly used in the clinical setting, it is a concentrate of autologous platelets as a source of growth factors, but the use of PRP has several drawbacks such as the liquid or gel consistency that causes the product to dissolve in surgical sites. Furthermore, the growth factor is usually released abruptly within the first two days of injection [5,6].

PRFM is a new generation platelet product consisting of a three-dimensional fibrin matrix. It is macroscopically denser and more elastic. The fibrin matrix entraps thrombocyte and the released growth factors, this resulted in the gradual release of growth factors to wound site over time. Previous studies had shown the use of PRFM in wound healing in which it concluded that there is an increase in the level of PDGF, VEGF. bFGF, and TGFβ on the first day after application which decreases gradually the next few days [[5], [6], [8]]. This characteristic is not present with PRP injection. Existing studies of PRFM in wound healing was done in chronic ulcer, however, the use of PRP and PRFM in skin graft healing was rarely studied.

This study aims to compare the use of platelet-rich plasma (PRP) and platelet-rich fibrin matrix (PRFM) in accelerating wound healing process of skin full-thickness skin graft (FTSG), by assessing polymorphonuclear neutrophils (PMN), macrophage, and fibroblast level using microscope evaluation also TGFβ1 and PDGF level using ELISA examination.

## 2 Materials and Methods

### 2.1 Animal model

This study was conducted at X, where 5 pigs (1 male and 4 females, aged 6–8 months, body weight around 27–40 kg) from a domestic breed were used (*Sus scrofa porcines* - *strain Landrace*). All the pigs included in this study had normal hematological profile, especially platelet counts. The number of samples was determined using the resource equation method, and ethical approval was obtained from Animal Care and Use Committee (ACUC).

During the research process; general anesthesia was administered to all the subjects followed by wound treatment, and administration of binder to protect skin graft. Before going under anesthesia, the pigs fasted for a minimum of 12 h, intramuscular atropine sulfate was injected as premedication. Anesthesia was done using Ketamine HCl 11 mg/kg, and xylazine HCl 0,2 mg/kg as a sedative, analgesic, and muscle relaxant. Anesthesia maintenance was achieved using isoflurane 1.5%. Lidocaine HCl 2% was used as local anesthesia for the area in which the skin graft was conducted.

### 2.2 FTSG

Using two rectangular specimens of full-thickness skin graft (FTSG), the samples were harvested on the back of the pigs’ body, which was the most ideal location to grow FTSG. These FTSGs were made using surgical blade no. 10 which is 9 cm × 3 cm each, the FTSG was then given a code. Each rectangular FTSG was signed with three circular sutures, for PRP, PRFM, and control.

### 2.3 PRP & PRFM preparation

**Fig. 1:**
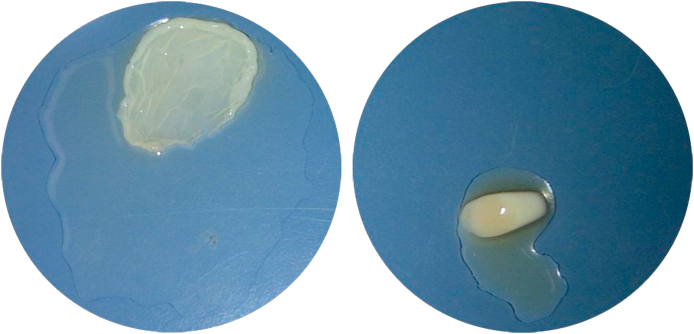
PRP (left) and PRFM (right) after the preparation process. The PRP are being prepared through mixing the gel layers (leukocyte and platelets) and plasma layers. PRFM are created through centrifugation of PRP.

ReagenKit tube from ReagenLab, Le Mont, Switzerland with a capacity of 8 mL of citrate blood was centrifuge at 1500 g for 5 min. This resulted in three layers, which include (from the bottom up); red blood cell, gel from the ReagenKit, and plasma. The gel layer (which is high in leukocyte and platelet) and plasma layer (platelet-poor plasma or PPP) were then mixed by slowly turning the tube back and forth three times. From this process, the mixture of gel and plasma layers was defined as PRP. The process of making PRFM was a continuation of PRP preparation, with 25 mM CaCl_2_ added into the PRP solution, followed by 1800 g centrifugation for 60 min. This PRFM creation method was a modification of Fibrinet method, which obtained a coin-shaped sheet PRFM with a diameter of 30 mm (see Fig. 1). For each step, this research used an automatic cell counter Celtac-α (Automated Hematology Analyzer MEK-6450, Japan) to count the number of platelets.

### 2.4 PRP & PRFM application

**Fig. 2:**
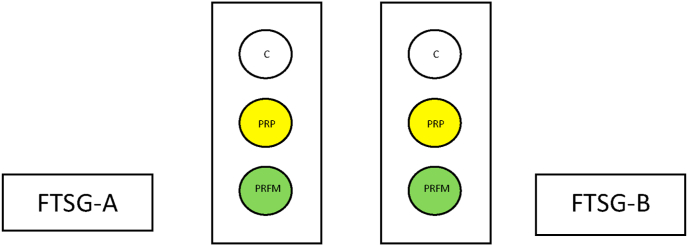
PRP and PRFM application in FTSG area. Each circular area is separated from one another to ensure that exact treatment effect on wound healing can be observed. C is for control (no administration of PRP or PRFM).

PRP infiltration and PRFM application are done prior to FTSG implantation. On separate circular areas on the pigs’ back (each with a diameter of 2.5 cm); PRP was injected into the bed area of the skin graft whilst PRFM was applied to the bed area of the skin graft. The third circular area for control was left without any PRP or PRFM (Fig. 2). All the areas were then given small incisions for blood drainage. The skin graft was then fixated by tight sutures for 7 days. Application of PRP and PRFM were done by the researcher whilst subsequent biopsy and analysis of samples are conducted by a research assistant who is blinded of the treatment allocation on each circular area.

### 2.5 Punch biopsy

On the 1st, 3rd, 7th, 14th, and 30th day, punch biopsy with a diameter of 0,6 cm was taken from each FTSG circular bed (control, PRP, PRFM). Punch biopsy result from the 1st and 3rd day shows that the FTSG and its bed are still separated, therefore it needed to be gathered via preparation in a paraffin block. Every punch biopsy was divided into two separate samples; for microscopic and ELISA evaluation.

### 2.6 Macroscopic evaluation

Survival rate of the skin graft was macroscopically evaluated on the 14th, and 30th day by assessing color changes of the skin from photographic documentation using Canon Ixus 900 Ti digital camera (Canon USA, New York, NY). A mark was made to distinguish each skin graft areas with different treatment. Photo results were then separated according to these marker before it’s processed and analysed using ImageJ software, where the color of each skin graft was assessed. Rate of differences in color (where darker color of skin means lower percentage of survival, and vice-versa) was used to indicate survival rate of the graft. Conversion of photograph results to ImageJ interpretation was done independently for each treatment area to prevent subjectivity and bias in assessment. Skin color that is closer to normal skin was interpreted as better survival.

### 2.7 Extracellular matrix evaluation (Pricosirius red examination)

This examination aims to assess the density of type 1 collagen. The assessment uses 2 polarizing glasses placed on top of the light source and slide containing the biopsy sample. The two polarizing glasses are positioned in such a way that a reddish orange color will be obtained which indicates the presence of type 1 collagen. The inflammation area is photographed, and the percentage of type 1 collagen density is measured using ImageJ software.

### 2.8 Microscopic evaluation

In the microscopic evaluation; the histological assessment was aimed to calculate PMN, macrophage cell population, and fibroblast cell proliferation. For PMN, macrophage, and fibroblast count; the biopsy result was tinted with hematoxylin-eosin and the cell counting was done using a microscope with 400*x* magnification. The calculation was done by measuring the cell per field of view (FOV). For PMN, the result is considered as positive when PMN fill out >50% of FOV, macrophage >10% of FOV, and fibroblast >10% of FOV.

Five FOV was examined in which the results are reported in 5 groups:

• 0 if 0 out of 5 FOV shows a positive result
• +1 if 1–2 out of 5 FOV shows a positive result
• +2 if 3 out of 5 FOV shows a positive result
• +3 if 4 out of 5 FOV shows a positive result
• +4 if 5 out of 5 FOV shows a positive result

The same grouping mechanism was applied to macrophage and fibroblast.

### 2.9 ELISA evaluation

The other half of the punch biopsy result was used for ELISA evaluation. This evaluation aimed to count TGFβ1 and PDGF levels. These particular growth factors were chosen because of its high titer level especially when compared to other growth factors. This measurement was done using R&D MB 100B for TGFβ1 and Antibody Online ABIN868875 for PDGF. The result was reported in pg/mg protein.

## 3 Result

Five pigs were used in this study, all of which are 6–8 months old and weighed between 27 and 40 kg. All of the pigs' hematological examinations were within normal range with platelet counts ranging between 567,400 ± 82,150/μL. One female pig was eliminated because it does not meet the study requirements (the pig died after transportation process from farm to faculty). Data from all the other pigs included were analysed for this study.

**Fig. 3:**
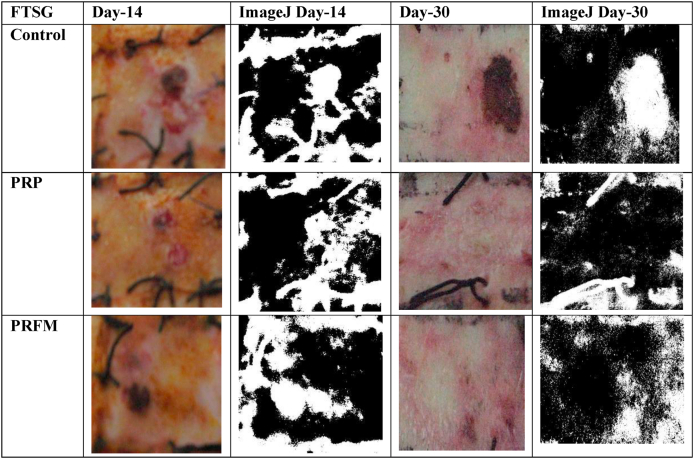
ImageJ analysis of FTSG survival at the 14th and 30th day. In each group, the image on the left shows graft documentation using a Canon Ixus 900Ti camera while image on the represents an ImageJ analysis of the graft. Black particles represent areas of good skin survival and are reported as a percentage of the total area analysed.

Macroscopic analysis using digital photograph and ImageJ software was conducted. The skin graft showed increased survival from day 14 and day 30 (Fig. 3). On day 14, the survival rate of FTSG for all treatments ranged from 48 to 50%, and on day 30 ranged from 65 to 67%. Overall, the 30th day shows better graft survival than the 14th day, but there were no significant difference in FTSG survival rates for all treatment groups in the four pigs.

**Fig. 4:**
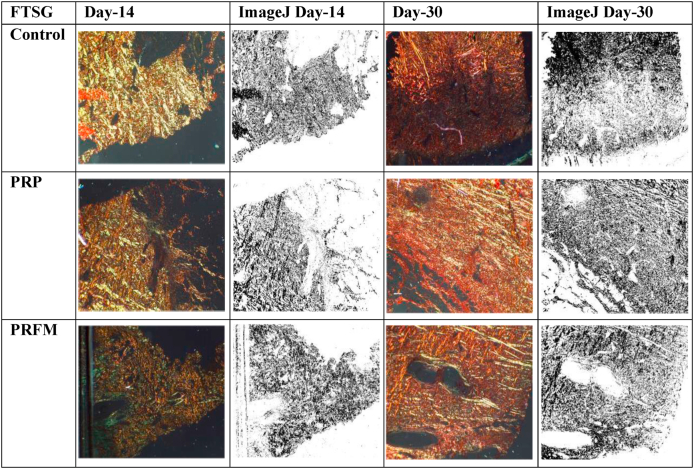
Picrosirius red staining and ImageJ analysis of collagen type 1 on day 14 (left) and day 30 (right) in FTSG-Control, FTSG-PRP, FTSG-PRFM. The black areas represent collagen type 1, generally appearing denser on day 30 compared to day 14. (For interpretation of the references to color in this figure legend, the reader is referred to the Web version of this article.)

Collagen type 1 increase were observed on day 14 and day 30 in all treatment groups. Even though statistically insignificant when compared with the other treatment groups, the highest value of collagen density in day 14 (25%) and day 30 (33%) was observed in FTSG-PRFM group (Fig. 4).

On microscopic (histological) examination, measurement was done by looking at the number of cells per field of view (FOV). Based on the physiological process, PMN will increase at the beginning of wound healing. Accordingly the result of the histological examination showed that PMN level was high on the 1st and 3rd days, with PRFM application shows higher PMN level compared to PRP and control. On following examinations after the 3rd day, the number of PMN continue to decrease until no PMN was observed on the FTSG area.

Theoretically, macrophages will start to appear in the inflammation stage of wound healing. On the 1st and 3rd day macrophage was not found in FTSG. An increase in the number of macrophages was observed on the 7th day, with PRFM application having the highest number of increases, followed by PRP, and then the control. The number of macrophages remained the same on the 14th day for PRP and PRFM application, but in the control areas, the number of macrophages had decreased. A decrease in macrophage was then observed on the subsequent follow up examinations in all treatment groups.

**Fig. 5:**
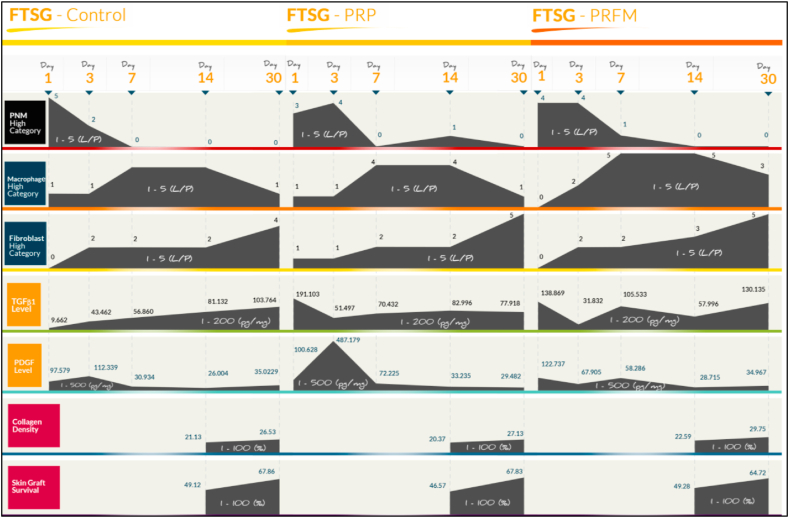
Summary of wound healing process showing results of macroscopic, microscopic, extracellular matrix, and ELISA evaluations from FTSG-control, FTSG-PRP, and FTSG-PRFM on day 1, 3, 7, 14, and 30.

In the process of wound healing, fibroblast will appear in the proliferation phase. Fibroblast was not detected on the 1st and 3rd day but was then observed on the 7th day, in which the highest level of fibroblast was observed in the control area, followed by PRFM. Areas with PRFM application on FTSG showed an increasing number of fibroblasts until the 30th day. Whereas fibroblast in the control area had already decreased on the 14th day, before increasing again on the 30th day. On PRP application, there was no significant increase of fibroblast until the 14th day, followed by a sharp increase on the 30th day (Fig. 5).

Growth factor measurement using ELISA shows their results in units of pg/mL, which were then converted into pg/mg protein. Upon examination, general findings show a very high level of TGFβ1 on the 1st day, both in PRP and PRFM application. When directly compared to each other; the TGFβ1 level from PRP application was higher than that of the PRFM. In both groups, a sharp decrease of TGFβ1 was seen on the second measurement (3rd day) followed by a visible increase on the 7th day, especially on the PRFM application. On the 30th day, TGFβ1 level increased in all groups. In contrast to TGFβ1, the increase in PDGF was only seen on the 3rd day. The only significant increase came in the PRP application. On the next measurement, no significant increase of PDGF was seen (Fig. 5).

## 4 Discussion

Pigs were chosen because of the similarity of their skin to humans; from the structures of epidermal, dermal, and skin color. However, with the usage of animal subjects, hygiene issues had been an obstacle in this study. It was solved by putting binder bands on the pigs, which were continuously replaced with new and clean binder bands after each biopsy schedule.

PRP was prepared using the RegenLab kit in which 8 mL of porcine blood was needed to produce PRP. It was centrifuged to produce 3 separate layers; red blood cells, gel, and plasma. PRP is the plasma that is located closer to the gel. The resulting PRP is around 6 mL with a thrombocyte concentration of 1,0 × 109 platelet. It is reported that during the making of PRP, 75% of platelet was missing, it might be caused by the platelet getting stuck in the plasma or red blood cells layer. PRFM was made by adding CaCl2 to the PRP solution followed by another centrifugation. After centrifugation PRFM was obtained in a coin-shaped matrix. There was a minimum residue of platelet in the PPP layer (0/mL) which means that all platelet was entrapped in the fibrin matrix.

The wound healing process started with coagulation activation and the inflammation process where platelets are activated to form a platelet plug. TGFβ1 and PDGF are produced from the platelet activation, which would then activate macrophages and stimulate fibroblast migration to the inflammation area. Once the macrophage is activated, it will also produce TGFβ1 and PDGF [[8], [9], [10], [11]]. Fibroblast on the other hand would also produce TGFβ1. The two growth factor that is being measured in this study is thus an accumulation of the overall healing process.

It was suspected that TGFβ1 and PDGF levels will be higher in the PRFM application, but these levels were observed to be higher in the PRP application. This occurs due to the activation of platelets by exogenous factors at the preparation time. It's suspected to have originated from the venipuncture procedure, the use of pipette, or the centrifugation process. Activated platelet will secrete α-granules, which in turn secrete growth factors (including TGFβ1 and PDGF) and other components. In the creation of PRFM, an extra step of centrifugation was conducted which causes the protein to remain in the plasma. As a result, the PRFM did not contain many growth factors (TGFβ1 and PDGF). However, it should be noted that TGFβ1 and PDGF have a short half-life, which means that PRP is not necessarily better than PRFM in accelerating wound healing because of its higher level of growth factors.

High TGFβ1 level on FTSG with PRFM application was found on the 1st and 3rd days. It is consistent with the high PMN count on the first days that slowly decreases on the following days, in exchange for the rise of macrophages. After macrophage infiltration to FTSG, TGFβ1 level rises again and remains high until 30th days following the high level of fibroblast [3,10].

The macrophages in the normal wound healing process started to decline on the 30th day as the inflammation process comes to an end. A high level of macrophages on FTSG with PRFM application shows that inflammation persists, resulting in a high TGFβ1 level. Compared to TGFβ1, PDGF only rises on the 1st day before starting to decline slowly the following days. PDGF is only produced by macrophages and fibroblasts when it's needed for fibroblast migration and proliferation [3,10].

In PRP application, PMN infiltration peaked on the 3rd day, followed by the rise of PDGF. PDGF is used for macrophage migration, yet in PRP application, macrophage level was still low on the 3rd day. Macrophage and fibroblast started to rise on the 7th day which causes an increase in TGFβ1. However overall macrophage and fibroblast level on PRP application was not as high when compared to PRFM [3,10].

In control FTSG, TGFβ1 and PDGF levels are not as high as PRP and PRFM application. TGFβ1 level still increases until the 30th day. The probable explanation is that TGFβ1 is still needed to recruit more fibroblasts. This shows that PRP and PRFM application in FTSG accelerates wound healing [11,12].

A high level of PMN on the 1st and 3rd days was seen in all treatments, these events are following the physiology of wound healing. As acute inflammatory cells, PMN is the first defense to carry out microbes or foreign objects. PMN in PRP application peaked on the 3rd day, while in PRFM application it peaked on the 1st day and remains high until 3rd day. This shows PRFM gives a better response in acute inflammation. PMN started to decrease on the 7th day per the normal wound healing process. After this process, PMN's role is replaced by the macrophage. In this study, it had been observed that macrophage level started to rise on the 7th day, after the decrease of PMN [3,14].

In contrast to PMN, macrophages do not only act as phagocytes but also as regulators that release cytokines and immunomodulatory factors. Growth factors (TGFβ1 and PDGF) that are produced by macrophages are playing important role in angiogenesis, migration of fibroblast, proliferation of fibroblast, collagen production, and wound contraction [10,13,14]. This study showed that both PRP and PRFM optimized wound healing, proven from high levels of macrophage in PRP and PRFM compared to control. PRFM application had the highest level of macrophage among all groups.

Fibroblast rises from the 3rd day until the 30th day. PRP and PRFM FTSG have a higher level of fibroblast compared to control. It shows that PRP and PRFM application increases fibroblast proliferation and promotes faster wound healing. PRFM application had the highest level of fibroblast proliferation.

Whilst macroscopic and extracellular evaluation showed no statistically significant difference between the effectiveness of PRP and PRFM, both evaluations had shown that using PRP and PRFM had a positive effect on the healing process. PRFM had also shown a slightly favorable result in extracellular evaluation by having more type 1 collagen formed on day 14th and day 30th. When correlated to the wound healing process, this finding can be clinically significant. In the proliferation phase, fibroblasts synthesize type 3 collagen. In the maturation phase, type 3 collagen will be replaced by type 1 collagen. Type 1 collagen will then stabilize wound healing areas so that it can eventually return to normal skin tissue [10]. The formation of additional type 1 collagen in PRFM (and to some extent in PRP when compared to control) might be representing that an effective wound healing process is taking place.

Summarizing from all the discussions above, it appears that the role of PRFM and PRP as autologous platelet preparation both accelerate wound healing. Furthermore, our observation also finds that PRFM application to FTSG resulted in a significantly higher level of PMN, macrophage, and fibroblast compared to PRP application. The findings in this study strengthen the concept of platelet concentrate usage in wound healing. It is also a practical proof of concept that PRFM can improve healing and the taking process of skin graft to the bed. It is clinically relevant, especially when conducting skin graft for patients with systemic comorbidities that may interfere with the skin graft healing process. With its efficacy proven; future studies would need to look upon side effects, complications, and general safety of using platelet concentrate to improve wound healing in humans.

Key limitations noted in this study were the fact that it only looked upon the usage of PRFM in skin graft on healthy animal test subjects. Conditions where a skin graft is deemed necessary (skin loss due to severe burns, chronic diabetic ulcerations, necrotizing fasciitis) involve different pathophysiology which may slightly interfere with the efficacy (or lack thereof) of PRFM in the skin graft healing process. The decision to administer PRFM in skin graft should be made on a case-by-case basis; with full consideration of patient's cases and conditions.

## 5 Conclusion

The role of PRFM in improving and accelerating the process of wound healing in full-thickness skin graft is better than PRP due to the higher number of PMN, macrophage, and fibroblast.

## Ethical approval

This study had been approved (05–2012 RSH-IPB) by the Animal Care and Use Committee of the Veterinary Teaching Hospital Institut Pertanian Bogor.

## Sources of funding

No sponsor had been involved in conducting this study.

## Author contribution

Dr. dr. Mirta Hediyati Reksodiputro, Sp.THT-KL(K) – Study concept, design, ethical submission, data collection, data analysis and interpretation, writing the paper.

dr. Alida Roswita Harahap, DMM, SpPK(K), PhD – Study concept, design, lab examination, data analysis and interpretation.

dr. Nurjati Chairani Siregar, Sp.PA(K), MS, PhD – Study concept, design, lab examination and data analysis.

drh. Safarina G. Malik MS, PhD – Study concept, design.

Prof. Dr. dr. Jenny Bashiruddin, Sp.THT-KL(K) – Study concept, design.

Prof. dr. Muhammad Thaufiq Siddiq Boesoirie, Sp.THT-KL(K) – Study concept and design.

Dr. dr. Dini Widiarni Widodo, Sp.THT-KL(K), M.Epid – Study concept, design.

Dr. dr. Sandi Iljanto, MPH – Data analysis and interpretation.

Prof. drh. Dondin Sajuthi, PhD – Study concept and design, provision of animal samples, data collection.

Dr. dr. Lugyanti Sukrisman, Sp.PD-KHOM – Sudy concept, design.

dr. Mikhael Yosia, BMedSci, DTM&H – Data analysis and interpretation, writing the paper and submission.

## Registration of Research Studies

The research doesn’t involve human participant.

## Guarantor

Dr. dr. Mirta Hediyati Reksodiputro, Sp.THT-KL(K).

## Provenance and peer review

Not commissioned, externally peer reviewed.

## Declaration of competing interest

All the authors declare that here is no conflict of interest in writing this paper.
